# 

**Published:** 1920-08-21

**Authors:** 


### TUBERCULOSIS NOTES.

### Essex Insurance Committee Meeting.

The finance sub-committee reported receipt of communications from the Ministry of Health regarding the rate of war bonus to be paid to practitioners for 1919 in respect of the increased cost of living, ranging from 30 per cent. of the capitation fees for treatment in cases where the total net professional annual income did not exceed £500, down to 15 per cent. where it was over £1,200; in both instances up to a maximum of £300. Some other concessions are also authorised. The County Council has "earmarked" a site for a county sanatorium, but nothing further has been done in the matter, the bargain as to the purchase of the land not having yet been struck. Criticisms of the results of the anti-tuberculosis scheme were made; the war has abundantly shown everywhere that the view of the genesis of tuberculosis which was fashionable in 1914 is now somewhat falsified.

### Association for Prevention of Tuberculosis.

The provisional programme for the seventh annual conference of the above body has just been issued, which it is proposed to hold in the Central Hall, Westminster, on October 16 next and the two following days. The subject to be considered is the completion of tuberculosis schemes in various relations. Dr. Addison, the Minister of Health, will touch on the activities of his own Ministry and of local authorities and insurance committees in an opening address, whilst Pensions Boards and general practitioners will form the subjects of addresses by Sir Robert Philip, Dr. Biggs, and others. The Red Cross and other voluntary agencies and the training of doctors and nurses will be considered on the second and third days by Sir Arthur Stanley and Sir Sims Woodhead. It is expected that leading French and Italian tuberculosis workers will be present, whilst evening social functions in connection with the conference are already arranged or in process of arrangement.

### Tuberculosis Schemes.

Extensive arrangements for treating tuberculosis continue to be mooted, and to be adopted and set in hand. In Hertfordshire there has been resolved upon the purchase of a mansion and 112 acres of land at a cost of £25,000, despite some opposition. It was stated that there are in the county 400 tuberculous patients, including 200 ex-soldiers, and that the number waiting for assistance amounts to thirty-two. In the city of Nottingham the Medical Officer of Health has made a report as to additional measures required. He thinks the time has come for making a systematic and well-organised effort to establish farm colonies and other open-air settlements for the after-treatment of persons with controllable phthisis who are willing to co-operate for their own good and that of others in similar circumstances. At Bulwell he suggests an institution for tuberculous children, part of the accommodation to replace a small children's sanatorium already in existence, and part to constitute a hospital for surgical tuberculosis. Until "indefinitely continuous" open-air and other appropriate treatment and occupation is provided for the cases of consumption amenable to treatment, so long will they continue to lapse and relapse, to their own hurt and that of the general community. The Warwickshire and Coventry Joint Committee, after a long discussion, carried by a majority of five votes to two a proposal to expend approximately £100,000 on the sanatorium treatment of tuberculosis.

August 16, 1920. "A WORKER."

### Gifts and Bequests.

UNIVERSITY COLLEGE HOSPITAL has been left £150 by Mr. Henry Lyne, of Portland Place, W.

THE Hospital for Sick Children, Great Ormond Street, has, failing certain family trusts, been left half the residue of the estate of Mr. F. W. Hasluck.

MEMBERS of the London Stock Exchange have collected £2,500 towards Lord Denbigh's appeal for £200,000 for the Royal National Orthopædic Hospital.

SIR JOHN THOMAS, a paper manufacturer, of Wooburn, Bucks, has left £1,000 each to the London Temperance Hospital, the High Wycombe Hospital, and the Marlow Cottage Hospital.

SIR FREDERICK COOK, Bart., Chairman of Cook, Son and Co., of St. Paul's Churchyard, left £15,000 to any London hospitals or to any central funds for the benefit of London hospitals as his executors shall determine. We are informed that this sum has been allocated by the executors to King Edward's Hospital Fund for London.

MR. T. W. TAYLOR, of Repton, has left £1,000 to the Derbyshire Royal Infirmary to endow a bed, and £500 to the Derbyshire Children's Hospital.

GUY'S HOSPITAL has been left £1,000 to endow a bed by Mr. A. C. Cole, of Newbury, Bucks.

THE ESSEX COUNTY HOSPITAL, Colchester, has been left £100 by Mrs. Wratislaw, of Colchester.

DAME E. L. LIBERTY, widow of Sir A. L. Liberty, of the Regent Street firm, has left £250 to the Royal Bucks Hospital, Aylesbury.

BY the will of the late Sir George Watson McAlpine, of Broad Oak, Accrington, Lancashire, the Victoria Hospital, Accrington, is left the sum of £1,000.

MR. J. H. HEWITT, of Stow-on-the-Wold, Gloucestershire, has bequeathed £500 to the Retford Hospital for a Hewitt Sanatorium Fund, and £500 similarly to the Bourton-on-the-Water Cottage Hospital. He has left also £200 to St. Mark's Hospital, City Road.

### A Child's Desire to Help King's College Hospital.

Elsie Bird, of 4 Oakfield Road, Anerley, S.E., a girl of about twelve years of age, being very anxious to help on the work of King's College Hospital, Denmark Hill, decided to save all the farthings given to her and give them to the hospital. This little donor brought the result of her savings to the hospital on Monday last, and when counted out it was found that the farthings amounted to 6s. 6d. For this welcome gift, Lord Hambleden, the Chairman, and the Committee of Management have conveyed to Elsie Bird their grateful thanks for her gift and their appreciation of the motive which prompted it. It is to be hoped that many others will follow the example of this little donor.

### Capetown's Hospital Needs.

THE ADMINISTRATOR'S SCHEME.

One of Capetown's urgent needs is an extensive and fully equipped hospital for acute cases, for the Cape Hospital Board reports that about a hundred cases a month are reluctantly refused admission to the institutions under its control. The Somerset Hospital and the existing suburban hospitals are inadequate in size and incomplete in equipment. Sir Frederic de Waal (the Cape Administrator) has drawn up a comprehensive scheme for the relief of all types of patients. Now that the community has approved his proposals, within a very few years Capetown should possess a chain of hospitals and homes. An early appeal will be made to the people to support the generous offer of the Provincial Administrator.

### The Value of the Emoluments of Infirmary Officers.

The Association of Poor-Law Unions and Parishes of London and Greater London (London Guardians' Association) has considered the question of the value placed on the emoluments of medical officers, matrons, nurses, etc., who have board and lodging provided. In view of the lessened purchasing power of money, the values previously placed on emoluments, viz., £100 per annum for "chief" officers and £52 for subordinate officers, are much below the actual cost if these officials lived outside the institution; and, as the superannuation allowance is based on salary, plus value of emoluments, it is obvious that an "indoor" officer who retires at the present time will not be in as good a position as an outdoor officer whose retiring allowance is based on salary, plus the *full* war bonus.

The Guardians' Association has now submitted its recommendations to the various Boards of Guardians, and it is hoped that these proposals will be uniformly adopted for the Metropolis :—

Principal officers' emoluments it is proposed to value at £200 per annum; subordinate officers' emoluments at £100 per annum.

The amounts are made up as follows : Principal officers—Board and lodging, £128; washing, £12; attendance, £60 = £200. Subordinate officers—Board and lodging, £78 per annum; washing, £9; uniform, £13 = £100.

### Insurance Against Unemployment.

Under the Unemployment Insurance Act, which will come into operation on November 8, substantially all persons for whom Health Insurance contributions have now to be paid, except outworkers and persons employed in agriculture and private domestic service, will have to be insured. The employees of local authorities and persons with rights under a statutory superannuation scheme are also excepted under certain conditions, but generally the exceptions include only persons who are excepted from the Health Insurance Acts. Workpeople over seventy will be insurable, except in the case of old-age pensioners. The contributions in the case of men of eighteen and over is 4d. each from employer and employee; of women of eighteen and over 3½d. per week from the employer and 3d. from the employee; of boys of sixteen and under eighteen, 2d. from each; and of girls of the same age, 2d. from the employer and 1½d. from the employee. For every contribution paid in respect of men and women the State contributes 2d. and 1⅔d. respectively, and proportionate amounts in the case of boys and girls. The employers' and employees' contributions will be made by affixing special stamps to unemployment books. These will be obtainable, not through the Post Office as in the case of Health Insurance, but through the Employment Exchanges. The benefit will be at the rate of 15s. per week for men and 12s. per week for women. No benefit is payable for the first three days of unemployment; thereafter it is payable for a maximum of fifteen weeks in any insurance year, with a certain limitation of the amount of benefit in proportion to contributions paid. Contributors who have made 500 contributions (or a smaller number if over the age of fifty-five on entry into insurance) will be entitled on reaching sixty years of age to a refund of their own contributions, less any benefit paid, together with interest. We dealt with certain aspects of this Act in our issue of June 19, p. 299.

Edmonton Board of Guardians have recently decided to adopt the principle of an eight-hour day for the nursing and domestic staffs at their hospital and the other institutions under the control of the Board.

### Matrons' and Sisters' Appointments.

Essex County Hospital, Colchester.—Miss Marion Pimm has been appointed sister of the men's surgical ward. She was trained at the Torbay Hospital, Torquay, where she was later staff nurse. She was also staff nurse at Brompton Hospital. Miss Byford has been appointed sister of the children's surgical ward. She was trained at the Essex County Hospital, later being sister of the men's surgical ward, and has been staff nurse at East London Children's Hospital, Shadwell.

Horton Infirmary, Banbury.—Miss Edith Storrs has been appointed night sister. She was trained at Rochdale Infirmary, where she was awarded the Gold Medal, and has been staff nurse and holiday night sister at the District Hospital, Mansfield. Miss Storrs has also done fever work at Ham Green Hospital, Bristol.

Guest Hospital, Dudley.—Miss K. Cooper has been appointed sister. She was trained at St. Bartholomew's Hospital, Rochester, and has been staff nurse at the Queen Victoria Cottage Hospital, Tonbridge.

Mary Hewetson Hospital, Keswick.—Miss Jessie M. Taylor has been appointed nurse-matron. She was trained at the Royal Infirmary, Chester, and has been matron of Longton Hospital and of the Ashburton and Buckfastleigh Cottage Hospital.

Montgomery County Infirmary, Newtown, Mid-Wales.—Miss Miriam Hicks has been appointed sister. She was trained at Dudley Road Infirmary, Birmingham, and has since been sister at Brighton Midwifery Training Home and sister-in-charge Ludlow Infirmary. She has also been engaged in private and district nursing.

Putney Hospital, Lower Common, S.W.—Miss Helen McEvoy has been appointed sister. She was trained at Kingston Infirmary, and has been at the Bermondsey Military Hospital for three and a-half years.

Romford Union Infirmary.—Miss Amy Phipps has been appointed deputy sister-matron. She was trained at St. George's-in-the-East Parish Infirmary, where she was later ward sister and relief night superintendent. She has worked as theatre sister and matron on war service. Miss Phipps holds the certificate of the Central Midwives Board.

Royal Hospital, Chesterfield.—Miss E. E. Birch has been appointed sister of the medical wards, and Miss A. A. Webb sister of the men's surgical ward. Both were trained at this institution. Miss Birch has since been sister of the military wards and sister of the female medical ward at the General Hospital, Wolverhampton. Miss Webb has been staff nurse and sister of the medical wards at the Royal Hospital, Chesterfield. She took her fever training at Seacroft Hospital, Leeds.

Southgate Hospital, Palmer's Green.—Miss Dorothea Webb has been appointed matron. She was trained at the General Infirmary and Children's Hospital, Kidderminster, and at the Western Fever Hospital, Fulham. She has been charge nurse at the Norwich Isolation Hospital, ward sister, theatre sister, and matron's assistant at the Victoria Hospital, Blackpool, and matron of the Langwith District Hospital, Derbyshire. Miss Webb is a member of the College of Nursing.

The Infirmary, Workington.—Miss Jean A. Shankland has been appointed matron. She was trained at the Royal Victoria Infirmary, Newcastle-on-Tyne, and has since been charge nurse at Tooting Fever Hospital, nurse at Northern Counties Nurses' Home, Newcastle-on-Tyne, and head sister at Workington Infirmary.

Victoria Hospital, Southend-on-Sea.—Miss Maud Elizabeth Parsons, R.R.C., has been appointed matron. She was trained at St. Thomas's Hospital, has been sister of the male surgical ward at Leicester Royal Infirmary, temporary night superintendent and ward sister at the North Evington Military Hospital, Leicester, and sister-housekeeper at Queen Alexandra's Hospital Home, Roehampton.

### St. Thomas's Hospital News.

### Medical Appointments.

H. B. Russell, Esq., M.B., B.S. Lond., M.R.C.P., resident assistant physician; L. N. Reece, Esq., M.B., B.S. Lond., F.R.C.S. Eng., resident assistant surgeon; W. B. Foley, Esq., O.B.E., M.B., B.S. Lond., resident orthopædic house surgeon.

### Scholarships.

The following Scholarships have been awarded in this Medical School:—Mr. M. W. P. Hudson, Entrance Science Scholarship, value £150; Mr. E. Thompson, Second Entrance Science Scholarship, value £60; Mr. D. P. Mulvany, Entrance Arts Scholarship, value £25; Mr. H. W. Bowen, Entrance Arts Scholarship, value £25; Mr. D. V. Davies, the William Tite Scholarship, value £25.

### Old Students' Dinner.

The St. Thomas's Hospital Old Students' dinner will take place at the Connaught Rooms, Great Queen Street, W.C., on Friday, October 1, 1920, at 7 o'clock for 7.30. The chair will be taken by Frederick Foord Caiger, Esq., M.D., F.R.C.P.

### The Book Market.

John Bale, Sons and Danielsson, Ltd.: "Air Sickness." By Crachet and Moulinier. 5s. net.

Ash and Co., Ltd.: "A Nursing Guide, 1920" (ninth issue). Edited by Matron, Guy's Hospital. 3s.

Henry Frowde and Hodder and Stoughton: "Cunningham's Manual of Anatomy." Vol. III. Seventh edition. 12s. 6d. net. "Common Infections of the Kidneys." By F. Kidd, M.B., B.C., F.R.C.S. 18s. net.

Baillière, Tindall and Cox: "War Against Tropical Disease." By Andrew Balfour. 12s. 6d. net.

W. Heinemann: "Selected Lectures and Essays, including Ligaments, their Nature and Morphology." Fourth edition. By Sir John Bland-Sutton. 15s. net. "Venereal Diseases: their Clinical Aspect and Treatment." By J. E. R. McDonagh, F.R.C.S. £3 3s. net.

Edward Arnold: "Cookery for Dyspeptics." By Dr. George Herschell. 2s. 6d. net.

G. Bell and Sons, Ltd.: "The Child-Welfare Movement." By Janet E. Lane-Claypon, M.D., D.Sc. 7s. net.

Borough of Nuneaton Education Committee: Annual Report of the School Medical Officer, 1919. Annual Report of the Medical Officer of Health, 1919.

County Borough of Warrington: Annual Report of the Medical Officer of Health. Annual Report to the Education Authority on School Hygiene, by G. W. N. Joseph, M.D., D.P.H.

